# Filamin A Is a Potential Driver of Breast Cancer Metastasis *via* Regulation of MMP-1

**DOI:** 10.3389/fonc.2022.836126

**Published:** 2022-03-11

**Authors:** Jie Zhou, Lvying Wu, Pengyan Xu, Yue Li, Zhiliang Ji, Xinmei Kang

**Affiliations:** ^1^ Department of Oncology, Xiang’an Hospital of Xiamen University, School of Medicine, Xiamen University, Xiamen, China; ^2^ State Key Laboratory of Cellular Stress Biology, School of Life Sciences, Xiamen University, Xiamen, China; ^3^ Department of Surgical Research, Universitätsklinikum Erlangen, Erlangen, Germany

**Keywords:** breast cancer, metastasis, FLNA, MMP-1, EMT

## Abstract

Recurrent metastasis is a major fatal cause of breast cancer. Regretfully, the driving force and the molecular beneath have not been fully illustrated yet. In this study, a cohort of breast cancer patients with locoregional metastasis was recruited. For them, we collected the matched samples of the primary tumor and metastatic tumor, and then we determined the mutation profiles with whole-exome sequencing (WES). On basis of the profiles, we identified a list of deleterious variants in eight susceptible genes. Of them, filamin A (FLNA) was considered a potential driver gene of metastasis, and its low expression could enhance 5 years’ relapse survival rate by 15%. To prove the finding, we constructed a stable FLNA knockout tumor cell line, which manifested that the cell abilities of proliferation, migration, and invasion were significantly weakened in response to the gene knockout. Subsequently, xenograft mouse experiments further proved that FLNA knockout could inhibit local or distal metastasis. Putting all the results together, we consolidated that FLNA could be a potential driver gene to metastasis of breast cancer, in particular triple-negative breast cancer. Additional experiments also suggested that FLNA might intervene in metastasis *via* the regulation of MMP-1 expression. In summary, this study demonstrates that FLNA may play as a positive regulator in cancer proliferation and recurrence. It provides new insight into breast cancer metastasis and suggests a potential new therapeutic target for breast cancer therapy.

## Introduction

Breast cancer has become the most common cancer and the main cause of cancer death in women. In 2020, there are an estimated 2.3 million new cases of breast cancers worldwide (11.7%), surpassing lung cancer (11.4%) in number for the first time (1). The global incidence rate and mortality rate of breast cancer are still increasing annually, and the increase in the lower sociodemographic index (SDI) countries is larger than that of higher SDI countries (2). The yearly-increasing cancer cases not only put heavy psychological pressure on patients but also raise great economic burdens to society and the country. In the new era of cancer therapy, breast cancers can be classified into four types according to the expression of estrogen receptor (ER), progesterone receptor (PR), human epidermal growth factor receptor 2 (HER2), and Ki-67 (3): Luminal A [ER+ and/or PR+, HER2−, Ki-67 < 14%], Luminal B [ER+ and/or PR+, HER2+; ER+ and/or PR+, HER2−, Ki-67 > 14%], HER2 positive (HER2+) [ER−, PR−, HER2+], and triple-negative breast cancer (TNBC) [ER−, PR−, HER2−]. The patients of specific cancer will receive individual therapy regimens to achieve the best therapeutic effect.

However, the tumor has the characteristics of heterogeneity, easy mutation of the genome, and strong adaptability to the external environment changes, which make it insensitive or resistant to various drug treatments and vulnerable to local recurrence or distal migration. Previous studies showed that over 25% of early breast cancer patients had metastases at the time of initial diagnosis (4), and about 30% of them would develop metastatic breast cancer in the future (5). The clinical outcome of breast cancer depends on the biology, extent, and location of metastasis. The luminal breast cancer has a higher propensity to develop bone metastases, while TNBC tends to metastasize to the lungs and brain (6, 7). Although the 5-year survival rate of breast cancer is increasing year by year, drug resistance, recurrence, and metastasis are still urgent problems in the treatment of cancer.

The occurrence and development of breast cancer are the results of the interaction of genes and environment, and the effect of the environment can also be manifested through genetic or epigenetic changes (8). Mark et al. found that *BRCA* plays an important role in breast cancer metastasis. *PALB2*, a key partner of *BRCA1/BRCA2*, was involved in DNA damage repair and tumor suppression activity; thus, its mutation can lead to increased susceptibility to breast cancer (9). In addition, the Max team found that loss of *p53* in cancer cells promoted Wnt secretion and triggered neutrophil inflammation through stimulating tumor-associated macrophages to produce IL-1β (10). There is a causal relationship between neutrophils and metastasis, in which the high neutrophil-to-lymphocyte ratio could promote the metastasis of breast cancer and reduce the survival rate of patients (11). What is more, *PTEN* is a tumor suppressor gene (12) related to a variety of human cancers and a major negative regulator of the PI3K/Akt signaling pathway (13). Abdullah et al. found that inhibition of *PTEN* can promote the activation of the PI3K/Akt pathway and further control the proliferation and development of breast cancer stem cells (CSCs) (14). Although many valuable efforts have been made, the genetic driving force underlying the recurrence and distal metastasis of breast cancers largely remains unexplored.

In this study, we collected nine pairs of primary and recurrent tumors of breast cancer patients, determined the mutation profiles with whole-exome sequencing (WES), identified potential driver genes, and further validated them with both cells and animal experiments. We intended to provide new insights into breast cancer metastasis and suggest potential new therapeutic targets for precise breast cancer therapy.

## Results

### Identification of Potential Driver Genes to Breast Metastasis

**Figure 1:**
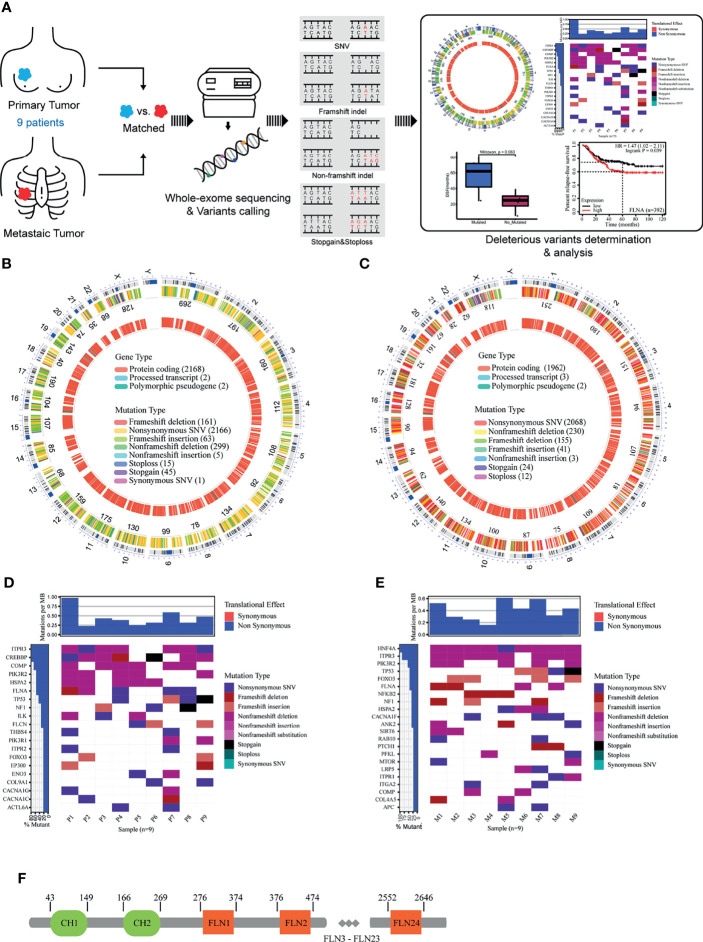
**(A)** Overview of study design. **(B)** The deleterious mutations in primary cancer. **(C)** The deleterious mutations in metastatic cancer. **(D)** Genes with deleterious mutations in at least two samples of primary cancer. **(E)** Genes with deleterious mutations in at least two samples of metastatic cancer. **(F)** The structure of human filamin A.

The WES of nine cohort patients (18 tissue samples) yielded a total of 47,407 high-quality and non-redundant somatic variants, including 27,845 single-nucleotide variants (SNVs), 16,679 insertions and deletions (indels), and 1,461 stopgain and stoploss mutations. To identify potential metastatic driver genes to breast cancers, we performed serial bioinformatics analyses (**Figure 1A**). The analyses were made based on an open assumption of the following: 1) the cohort patients may have different genetic backgrounds of metastasis (**Table 1**), 2) the metastatic driver gene mutations could be harmful (deleterious) to the cells, and 3) the deleteriousness of gene mutations would be a benefit to metastasis. Accordingly, we first narrowed down the whole mutation profiles to the harmful ones by integrating deleterious prediction results of multiple bioinformatics tools. A list of 2,755 deleterious mutations was obtained in the primary cancer samples consistently, including one synonymous SNV, 2,166 non-synonymous SNVs, 304 non-frameshift indels, 224 frameshift indels, 45 stopgain mutations, and 15 stoploss mutations. These deleterious mutations were distributed on all chromosomes except the Y chromosome, and the majority of them occurred in protein-coding regions (**Figure 1B**). Similarly, we obtained 2,533 deleterious mutations in the metastatic cancer samples consistently, including 2,068 non-synonymous SNVs, 233 non-frameshift indels, 196 frameshift indels, 24 stopgain, and 12 stoploss mutations. These mutations had similar chromosome distribution as those of primary cancer samples and were also located mainly at protein-coding regions (**Figure 1C**). These results manifest that primary tumors and metastatic tumors in this study have no genetic difference in general. Furthermore, we extracted the susceptible genes that have deleterious mutations in at least two samples of either primary tumor or metastatic tumor. The criteria eventually identified eight susceptible genes shared by primary/metastatic tumors; they were *COMP*, *FLNA*, *FOXO3*, *HSPA2*, *ITPR3*, *PIK3R2*, *NF1*, and *TP53* (**Figures 1D, E**). Literature surveillance manifested that these genes played multiple roles in cancers, such as cell growth, cell apoptosis, cell migration, and cell invasion (15–22).

**Table 1:** Detailed information of 9 breast cancer patients.

Sample ID	Age	TNM of initial diagnosis	ER	PR	HER2	DFS (months)
Patient1	56	T2N2M0	++	++	−	62
Patient2	56	T2N0M0	+	−	−	80
Patient3	46	T3N2M0	++	++	−	28
Patient4	44	T1N0M0	++	++	−	72
Patient5	68	T1N0M0	+++	−	++~++	22
Patient6	N.A.	T1N0M0	−	+	++	38
Patient7	N.A.	T1N0M0	−	−	+++	42
Patient8	58	T2N0M0	++	+	−	24
Patient9	30	T1N0M0	−	+	−	5

ER, estrogen receptor; PR, progesterone receptor; HER2, human epidermal growth factor receptor 2; DFS, disease-free survival.

N.A., Not Available.

**Figure 2:**
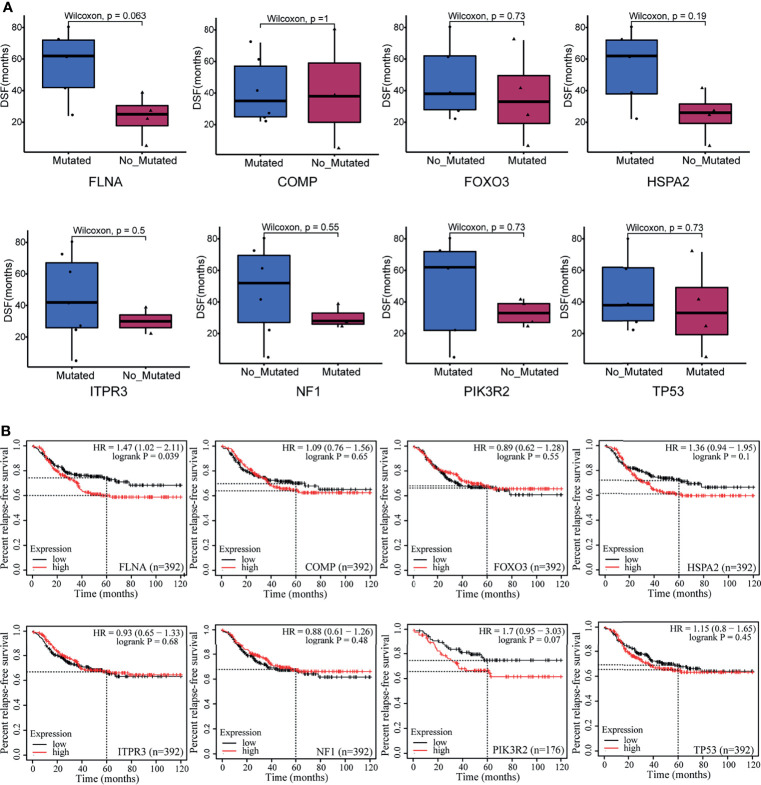
Exploring the relationship between eight susceptible genes and prognosis of breast cancer patients. **(A)** A progression-free survival (PFS) analysis on the deleterious mutants within the nine-member cohort. **(B)** The survival analysis was on basis of 392 triple-negative breast cancer (TNBC) patients from 55 independent experiments.

To further connect these genes with metastasis, we performed a progression-free survival (PFS) analysis on the deleterious mutants within the nine-member cohort (**Figure 2A**). Of the eight susceptible genes, only one gene (FLNA) exhibited significant change (two-tail unpaired Wilcoxon rank-sum test, *p* < 0.1) of PFS when the deleterious mutation occurred in primary cancer, which extended the PFS. In particular, the patients with deleterious mutations in FLNA in primary tumors had an average PFS value (n = 5, average PFS = 56 months) of about 2.5 times larger than that of those without the mutations (n = 4, average PFS = 23.3 months). Many of the deleterious mutations are located at the first few repeats of the immunoglobulin (Ig) domain (**Table 2**), causing the dysfunction of FLNA protein. In addition, we performed the survival analysis on basis of 392 TNBC patients from 55 independent experiments to examine the gene expression level of susceptible genes on metastasis, assuming that deleterious mutations would reduce the corresponding gene expressions. The result manifested that low expression of FLNA would significantly enhance 5 years’ relapse-free survival rate by 15% compared to that high expression group (**Figure 2B**). Putting all the data together, we speculate that FLNA could be one of the positive factors to breast cancer metastasis. Deleterious mutation of FLNA gene, particularly at its first few Ig repeats, would reduce its expression and thus resist metastasis.

**Table 2:** The deleterious mutations in FLNA of all samples.

Sample	Start	End	Ref	Alt	Type	AAchange	FLN repeat
Patient1-M	154360534	154360570	GCGGGCGGGGGAGCCCGCACTGCCTCCCTGCAGCCCC	–	Frameshift deletion	P1075fs	9
	154362486	154362491	TGTCAT	–	Non-frameshift deletion	831_833del	6
Patient2-M	154359888	154359891	TGGC	–	Frameshift deletion	A1274fs	11
Patient6-M	154362486	154362491	TGTCAT	–	Non-frameshift deletion	831_833del	6
Patient8-M	154362486	154362491	TGTCAT	–	Non-frameshift deletion	831_833del	6
Patient1-P	154361680	154361687	GCCAGACA	–	Frameshift deletion	V976fs	8
Patient2-P	154362486	154362491	TGTCAT	–	Non-frameshift deletion	831_833del	6
Patient4-P	154366374	154366374	C	T	Non-synonymous SNV	G388S	2
Patient7-P	154354220	154354220	C	T	Non-synonymous SNV	V1822M	16
Patient8-P	154352600	154352600	G	A	Non-synonymous SNV	S2144L	20

SNV, single-nucleotide variant.

### Cellular Consequence of Defected FLNA *via* Knockout Experiments

**Figure 3:**
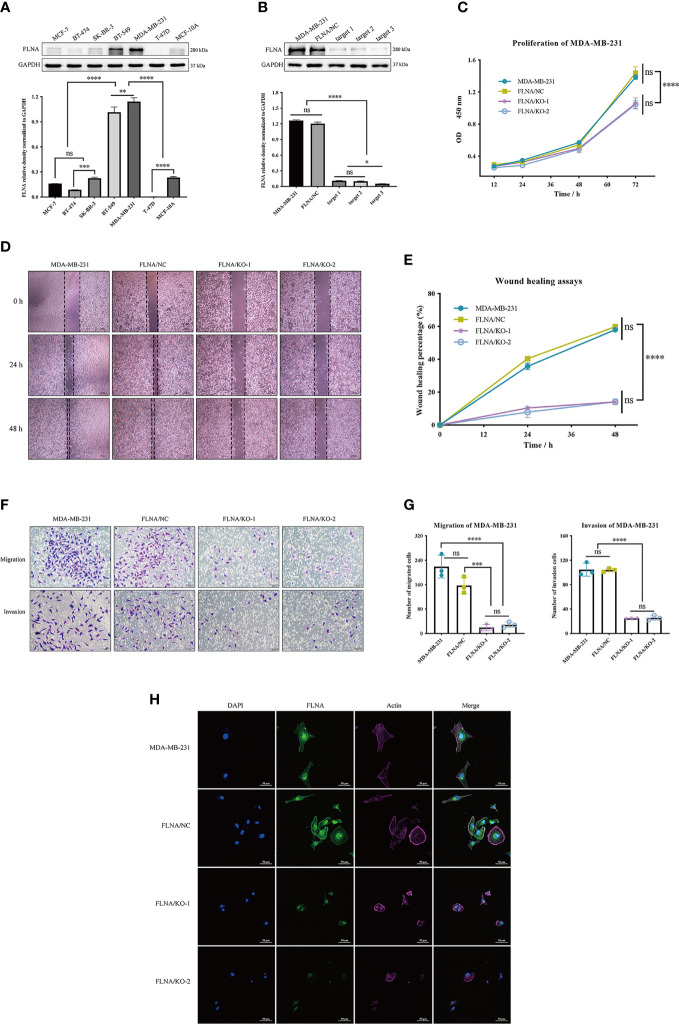
**(A)** The expression of FLNA in different breast cancer cell lines, followed by the quantitative and statistical analysis results of proteins in Western blotting. **(B)** FLNA knockout efficiency of MDA-MB-231 cells and the quantitative and statistical analysis results. **(C)** Cell Counting Kit-8 (CCK-8) assay presents the proliferation of different groups. **(D)** Wound‐healing assays (scale bar, 200 μm) were used to detect the migration abilities of the cells. **(E)** The wound‐healing percentage of different groups. **(F)** Transwell and invasion assays present the migration (scale bar, 50 μm) and invasion abilities of the cells. **(G)** The number of invaded MDA-MB-231 cells, FLNA/NC, and FLNA/KO cells. **(H)** Breast cancer cells (scale bar, 50 μm) with/without FLNA knockout were stained with fluorescein-phalloidin (pink) to visualize F-actin. DAPI was used for nuclear staining (blue). FLNA was stained in green. Data are presented as mean ± SD. The data shown are representative results of three independent experiments. *p < 0.05, **p < 0.01, ***p < 0.001, ****p < 0.0001, ns, no significance.

We examined the protein level of FLNA in breast mammary epithelial cells (MCF-10A) and different breast cancer cell lines mentioned in the *Material and Methods* with Western blotting. Comparatively, FLNA is highly expressed in MDA-MB-231 (**Figure 3A**). Hence, we constructed knockout cells of MDA-MB-231. Subsequent Western blotting validated the successful knockout of FLNA in different target cells (**Figure 3B**). Accordingly, we chose two knockout cell lines of MDA-MB-231, target 1 and target 2, namely, FLNA/KO-1 and FLNA/KO-2, respectively, for cell proliferation and migration assays. The results showed that knockout of FLNA caused a decrease of proliferation for 76.35% in FLNA/KO-1 and 75.61% in FLNA/KO-2 cells at 72 h (**Figure 3C**), and the wound healing capability of cells dropped 43.95% and 43.84% at 48 h, respectively (**Figures 3D, E**). Besides, the migration and invasion ability of FLNA/KO-1 cells decreased 91.17% and 87.06%, and the FLNA/KO-2 cells decreased 76.43% and 75.48%, respectively (**Figures 3F, G**). Comparatively, the negative control (NC) showed no significant difference from the wild-type MDA-MB-231 in all aspects of cell proliferation, wound healing, migration, and invasion. These results confirm that knockout of FLNA is not fatal to cancer cells; however, it can repress cell proliferation, migration, and invasion. Immunofluorescence (IF) assay showed that FLNA was mainly distributed in the cytoplasm and nucleus (**Figure 3H**). Compared with wild-type and NC group cells, FLNA/KO cells had smaller sizes and poor cytoskeleton development (**Figure 3H**).

### Knockout of FLNA Decreases Xenograft Tumor Growth and Metastasis

**Figure 4:**
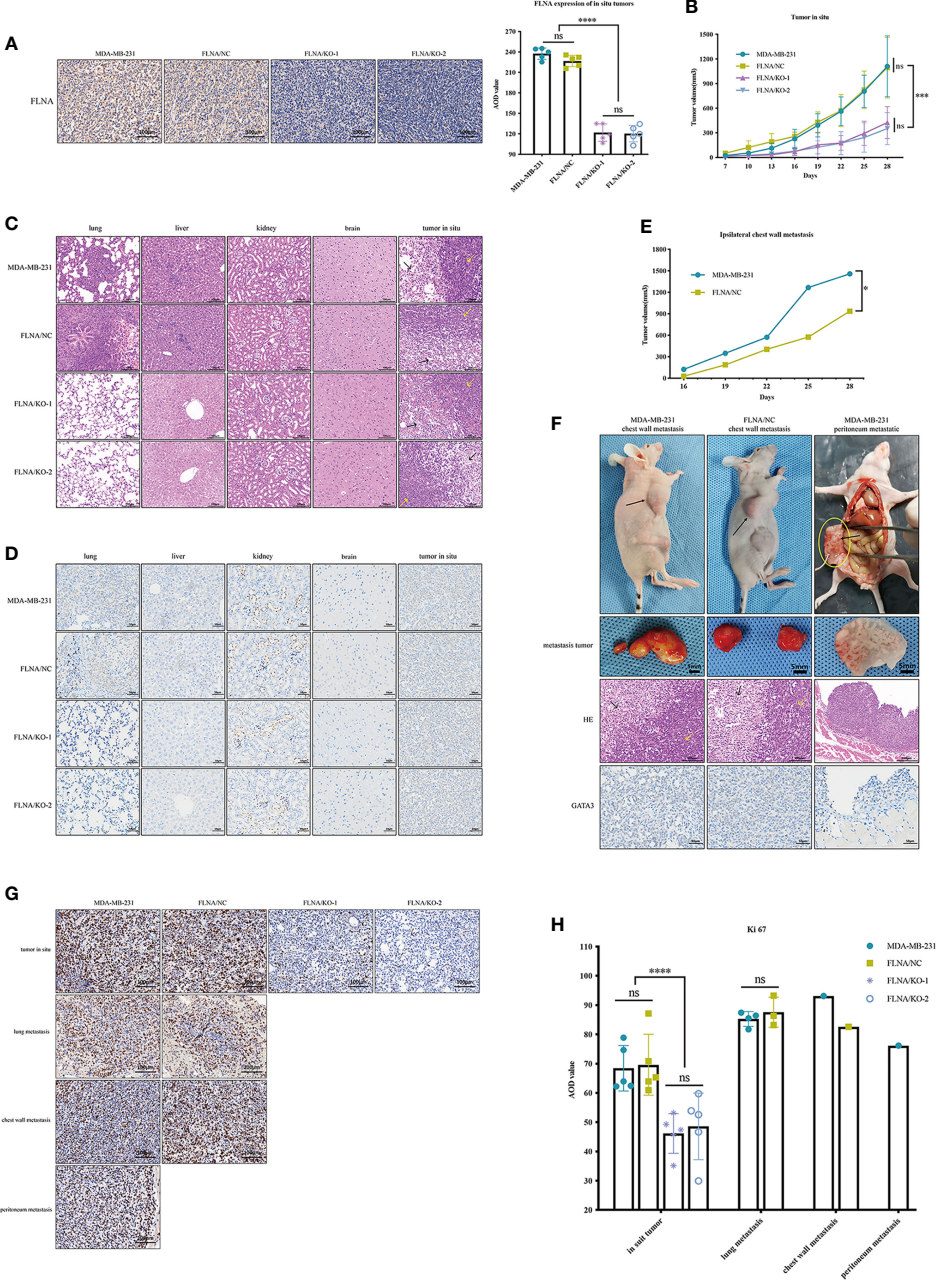
**(A)** Representative immunohistochemistry (IHC) images (scale bar, 100 μm) of tissue sections of *in situ* tumor from the four groups. FLNA was stained brown in cytoplasm and nucleus. Representative IHC images (scale bar, 100 μm) of tissue sections of *in situ* tumor from the four groups. FLNA was stained brown in cytoplasm and nucleus. Beside it is the average optical density (AOD) value of FLNA *in situ* tumor tissues. **(B)** The volume (mm^3^) of *in situ* tumor in each group was recorded every 3 days. **(C)** Representative H&E (scale bar, 100 μm) staining of tissue sections of different organs from the four groups (n = 5). The cells near the margin of *in situ* tumor are indicated by yellow arrows, and the center cancer cells were indicated by black arrows. **(D)** Representative IHC images (scale bar, 50 μm) of tissue sections of different organs from the four groups (n = 5). GATA3 was stained light brown in the nucleus, which was mainly expressed in the nucleus and often used for detecting the breast origin tumor. **(E)** The volume (mm^3^) of ipsilateral chest wall metastatic tumors of two mice in MDA-MB-231 and FLNA/KO groups. **(F)** Pictures of ipsilateral chest wall metastasis and peritoneal metastasis in nude mice in MDA-MB-231 and FLNA/NC groups. Black arrows indicate tumor location. In the H&E (scale bar, 100 μm) staining results, the marginal cells of metastatic tumor are indicated by yellow arrows, and the central cells are indicated by black arrows. GATA3 (scale bar, 50 μm) colored the nucleus light brown. **(G)** The expression level of Ki-67 in different tissues (scale bar, 100 μm). Ki-67 colored the nucleus brown. **(H)** The Ki-67 AOD value of different tumor tissues. Data are presented as mean ± SD. *p < 0.05, ***p < 0.001, ****p < 0.0001, ns, no significance.

To further study the functional role of FLNA, we used wild-type MDA-MB-231, FLNA/NC, FLNA/KO-1, and FLNA/KO-2 stably transfected cell lines to establish xenograft models. Each model had five repeated cases. We monitored the expression of FLNA in mouse *in situ* tumors and found that FLNA/KO groups decreased by 46.25% and 46.91% (**Figure 4A**). Compared to wild type and NC, the FLNA/KO mice had significantly slower tumor growth rate and smaller tumor volume (declined 61.72% and 68.30%, respectively) by 28 days (**Figure 4B**). The tumor volume of two cases with ipsilateral chest wall metastasis was recorded in **Figure 4E**. H&E stain of the xenograft tumor showed that there may exist two morphologies of cancer cells in the orthotropic tumor (**Figure 4C**): the cancer cells near the margin of *in situ* tumor were large, with obvious atypia large nucleus, common mitosis, and basophilic cytoplasm (indicated by yellow arrows). In contrast, the cancer cells in the center of *in situ* tumor were small or medium-sized, more consistent in shape, mostly round or oval, and loosely arranged and had fewer mitosis (indicated by black arrows). This phenomenon may be owing to the tumor growth exceeding the growth rate of the blood vessels providing nutrition, resulting in tissue necrosis or even liquefaction of some central tissues due to insufficient energy supply. We also observed that the morphology of lung metastatic cancer cells had large cells, rich chromatin, and common mitotic images. The liver metastasis cells from breast cancer were small and round, and the cell size and shape were consistent.

In addition, we demonstrated the immunohistochemistry (IHC) assay to monitor metastasis with the marker GATA3, which is often used for detecting urothelial or breast origin tumor. As GATA3 was positively related to ER and PR status (23), the nucleus of the MDA-MB-231 cell xenograft appeared to be light brown (**Figure 4D**). In summary, no local or distal metastasis was observed in the FLNA/KO groups. In contrast, one case of liver metastasis, four cases of lung metastases, and one case of ipsilateral chest wall metastasis were found in the wild-type MDA-MB-231 group; and one case of liver metastasis, three cases of lung metastases, and one case of ipsilateral chest wall metastasis were observed in the FLNA/NC group. H&E and IHC of chest wall metastasis and peritoneal metastasis are shown in **Figure 4F**. Similar to the H&E results of *in situ* tumors, the marginal cells of metastatic tumors were large, and nuclear atypia was obvious (which is indicated by yellow arrows). However, the central cells were small and loosely arranged. GATA3 colored the nucleus light brown, indicating that the tumor was of breast origin. Furthermore, we also detected the expression of Ki-67 in tumors *in situ* and metastases. The result manifested that Ki-67 is expressed low in the FLNA/KO groups and high in the other groups, suggesting a strong ability of cell proliferation (**Figures 4G, H**).

### FLNA Regulated the Expression of MMP-1

**Figure 5:**
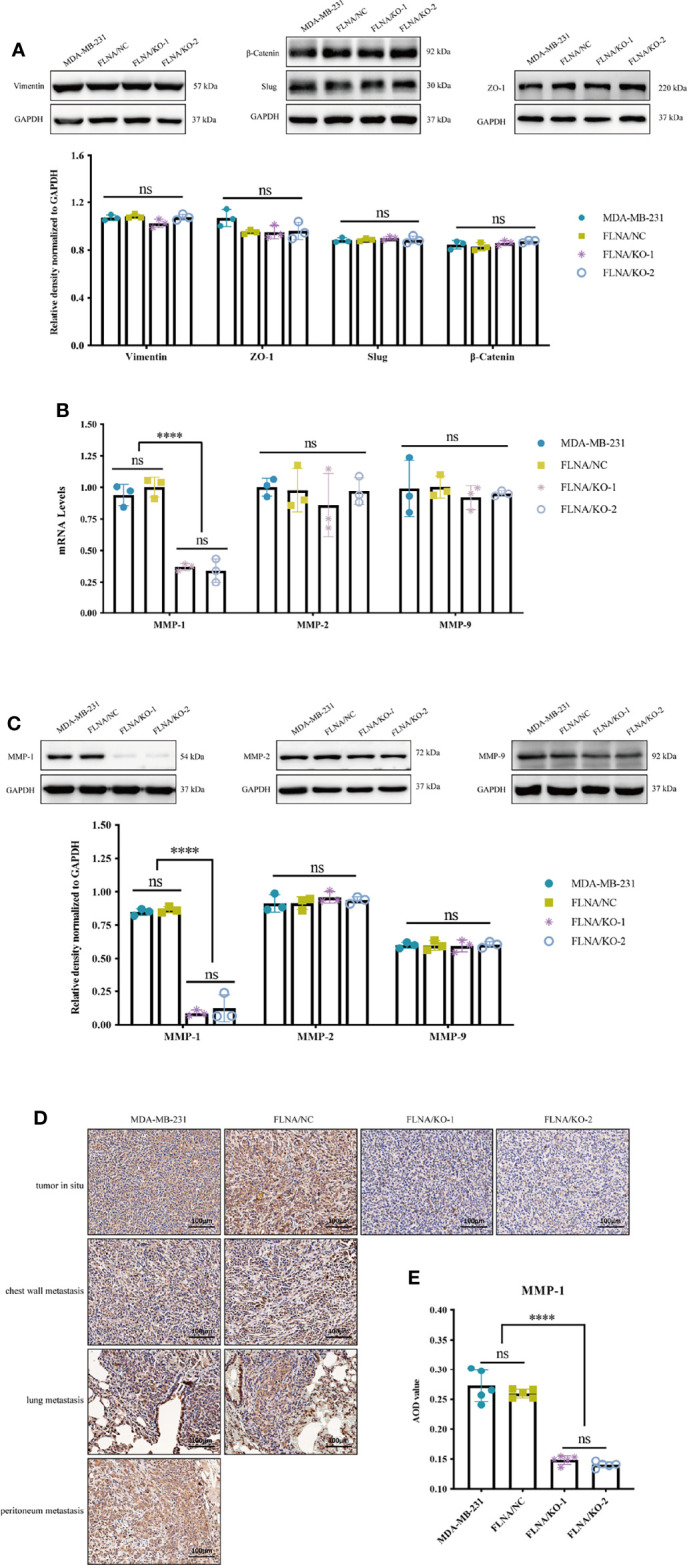
**(A)** Epithelial-to-mesenchymal transition (EMT)-related pathway protein expression level and the relative density normalized to GAPDH. **(B)** The mRNA expression level of MMP-1, MMP-2, and MMP-9 in different group cells. **(C)** The protein expression level of MMP-1, MMP-2, and MMP-9 in different group cells and the quantitative and statistical analysis results of proteins. **(D)** Expression of MMP-1 in different tissues (scale bar, 100 μm). **(E)** The MMP-1 AOD value of *in situ* tumor tissues in four groups. ****p < 0.0001, ns, no significance.

Previous studies have shown that tumor metastasis is closely related to epithelial-to-mesenchymal transition (EMT) (24) and extracellular matrix (ECM) (25), and they were recognized as critical factors in governing metastatic colonization. In the process of EMT, the cells showed decreased adhesion and increased motility, which led to metastasis of malignant tumor cells (26). ZO-1 is indispensable for tight junction formation and function (27), in which mutation can induce EMT (28). Slug is a widely expressed transcriptional repressor protein that, when combined with the integrin promoter, inhibits integrin expression and leads to decreased cell adhesion (29). β-Catenin can activate slug, which is related to tumorigenesis (30). Vimentin is highly expressed in a variety of tumors, which is closely related to promoting tumor growth, invasion, and poor prognosis (31). Therefore, we first detected the expression of EMT-related proteins and found that FLNA/KO had no significant effect on EMT (**Figure 5A**). Therefore, we concluded that FLNA may not affect the metastasis of breast cancer through the EMT pathway, and there may exist other ways. After that, we determined the mRNA levels of several conventional ECM components such as MMP-1, MMP-2, and MMP-9 in response to FLNA knockout with RT-qPCR. Interestingly, of these major ECM components, only MMP-1 decreased after FLNA knockout (**Figure 5B**). This result was further confirmed in protein level (**Figure 5C**). We detected the expression of MMP-1 *in situ* and metastatic tumors of breast cancer xenograft in mice, and we found that MMP-1 decreased by 44.23% and 47.23% in FLNA/KO-1 and FLNA/KO-2 groups, respectively (**Figures 5D, E**). These results indicated that FLNA could affect the metastasis of breast cancer cells by regulating the expression of MMP-1.

### Overexpression of MMP-1 Promotes Cell Growth and Migration

**Figure 6:**
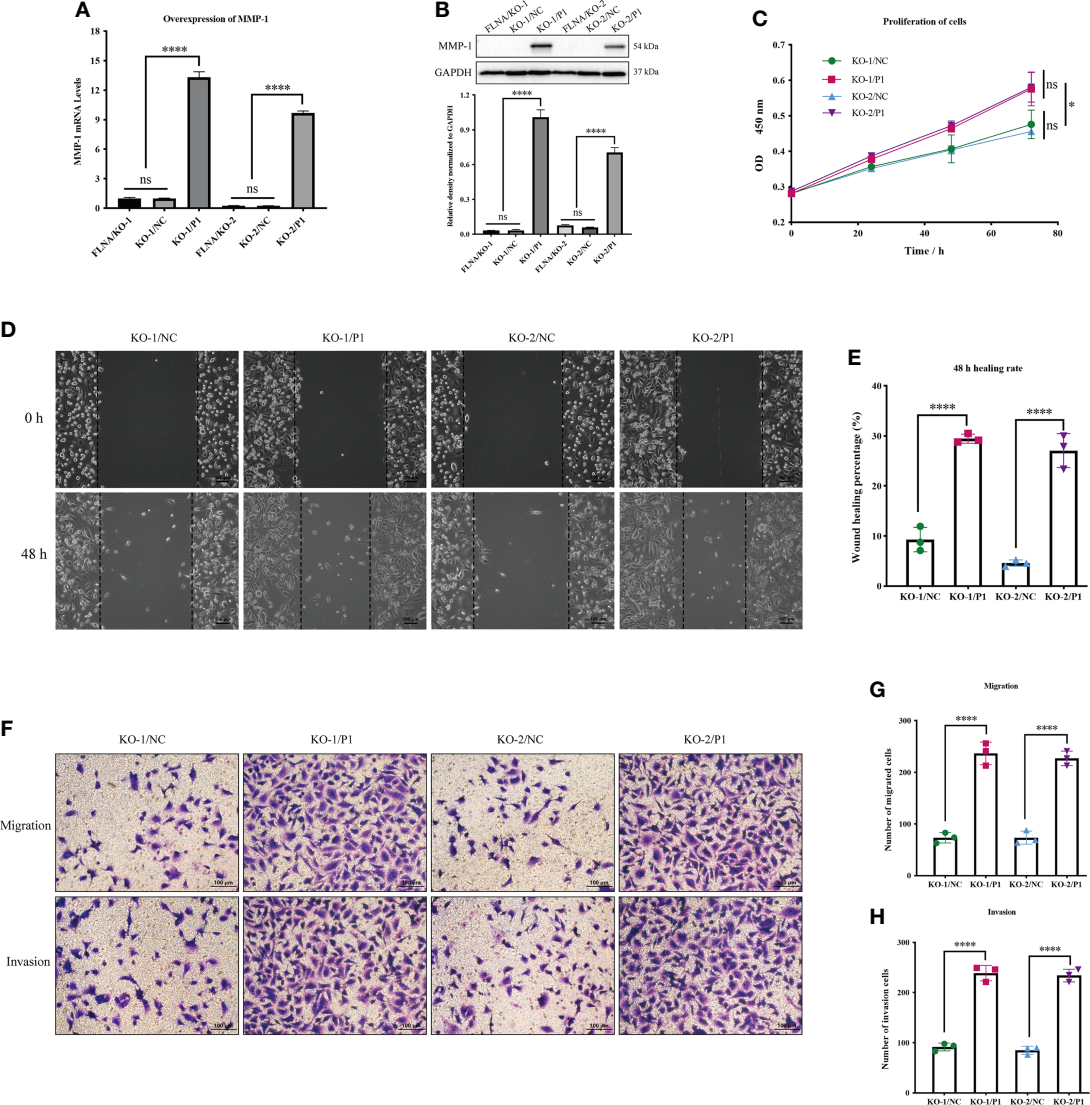
**(A)** The mRNA expression level of MMP-1 after transfection. **(B)** The protein expression level of MMP-1 after transfection. **(C)** Cell Counting Kit-8 (CCK-8) assay presents the proliferation of different groups. **(D)** Wound‐healing assays (scale bar, 100 μm) were used to detect the migration abilities of the cells. **(E)** The wound‐healing percentage of different groups. **(F)** Transwell assays present the migration and invasion abilities of the cells (scale bar, 50 μm). **(G)** The number of migration cells in different groups. **(H)** The number of invasion cells in different groups. *p < 0.05, ****p < 0.0001, ns, no significance.

We overexpressed MMP-1 in two FLNA knockout stably transfected cell lines, FLNA/KO-1 and FLNA/KO-2, in an attempt to explore whether MMP-1 can reverse the antitumor effect. We used PCR and Western blotting to monitor the transfection efficiency and expression level of MMP-1. PCR results showed that the overexpression efficiency of KO-1/P1 and KO-2/P1 was 13.3 times and 38.85 times higher than that of KO-1/NC and KO-2/NC, respectively (**Figure 6A**). Western blotting showed that the expression levels of MMP-1 in KO-1/P1 and KO-2/P1 were respectively 23.55 and 11.67 times higher than those in the NC (**Figure 6B**). Overexpression of MMP-1 could promote the proliferation of FLNA/KO cell lines, which increased by about 1.24 times at 72 h (**Figure 6C**). The wound healing capability of the two cell lines increased by 3.17 and 5.89 times at 48 h, respectively (**Figures 6D, E**). In addition, we also observed changes in migration and invasion. Transwell experiment showed that the number of cell migration of KO-1/P1 and KO-2/P1 was respectively 3.23 and 3.08 times higher than that of NC groups (**Figures 6F, G**), and the invasion ability was increased by 2.6 and 2.75 times (**Figures 6F, H**), respectively. These results suggest that overexpression of MMP-1 can reverse the antitumor effect of FLNA knockout to a certain extent.

## Discussions

Early studies reported that the genetic variants in *TP53* (32), *BRCA1* (33), and *EGFR* (34) could intervene in tumorigenesis and tumor development. Regretfully, none of these mutations were observed in this study. Instead, this study identified several novel deleterious variants likely associated with the recurrence of breast cancers in a small cohort. Of them, filamin A (FLNA) showed the most potential in regulating breast cancer metastasis and PFS, in particular in TNBCs. FLNA is a 280-kDa protein that can be cleaved into two fragments of 170 kDa (ABD + Rep.1–15) and 110 kDa (Rep.16–24). The latter one is near the C-terminal region, which can be further cleaved into a 90-kDa fragment (Rep.16–23, FLNA-C) (35). Previous studies had demonstrated FLNA could intervene in cancer development *via* promoting or inhibiting the expression of some genes. For instance, a metadata analysis on basis of 392 TNBC samples from 55 separate experiments suggested that low expression of FLNA could significantly enhance the 5-year relapse survival rate compared to that of high expression. A large-scale clinical study revealed that the overphosphorylation of FLNA Ser2152 was associated with a poor prognosis of hepatoma, which may be a potential prognostic biomarker of primary liver cancer (36). Bojan et al. found that microRNA-200c could reduce FLNA by inhibiting the transcription factors c-Jun and MRTF/SRF and thereby affect the polarization of breast cancer cells, resulting in the cell morphology changes and decreased motor ability (37). Another study showed that ADP ribosylation factors like 4C (Arl4C) could interact with FLNA rep.22 in a GTP-dependent manner to induce filopodium formation and promote cell migration (38). Therefore, we speculate that FLNA plays an important role in tumor metastasis. Although FLNA was reported to be highly expressed in cancers (39–41), its connection with breast cancer metastasis has not been well investigated previously.

In this study, we proposed that FLNA could be a positive factor in breast cancer metastases for the first time. The *in vitro* cell assays confirmed the fundamental function of FLNA as a scaffold in constructing the actin cytoskeleton. Knockout of FLNA did not sacrifice cells; however, it impaired cell cytoskeleton and largely reshaped the cells to a smaller size. Thereby, the proliferation, migration, and invasion of cancer cells were significantly weakened. The *in vivo* xenograft mouse model further consolidated that knockout of FLNA largely repressed the local and distal metastases of transfected tumors. All shreds of evidence strongly support that FLNA is a positive driver gene of breast cancer metastasis.

**Figure 7:**
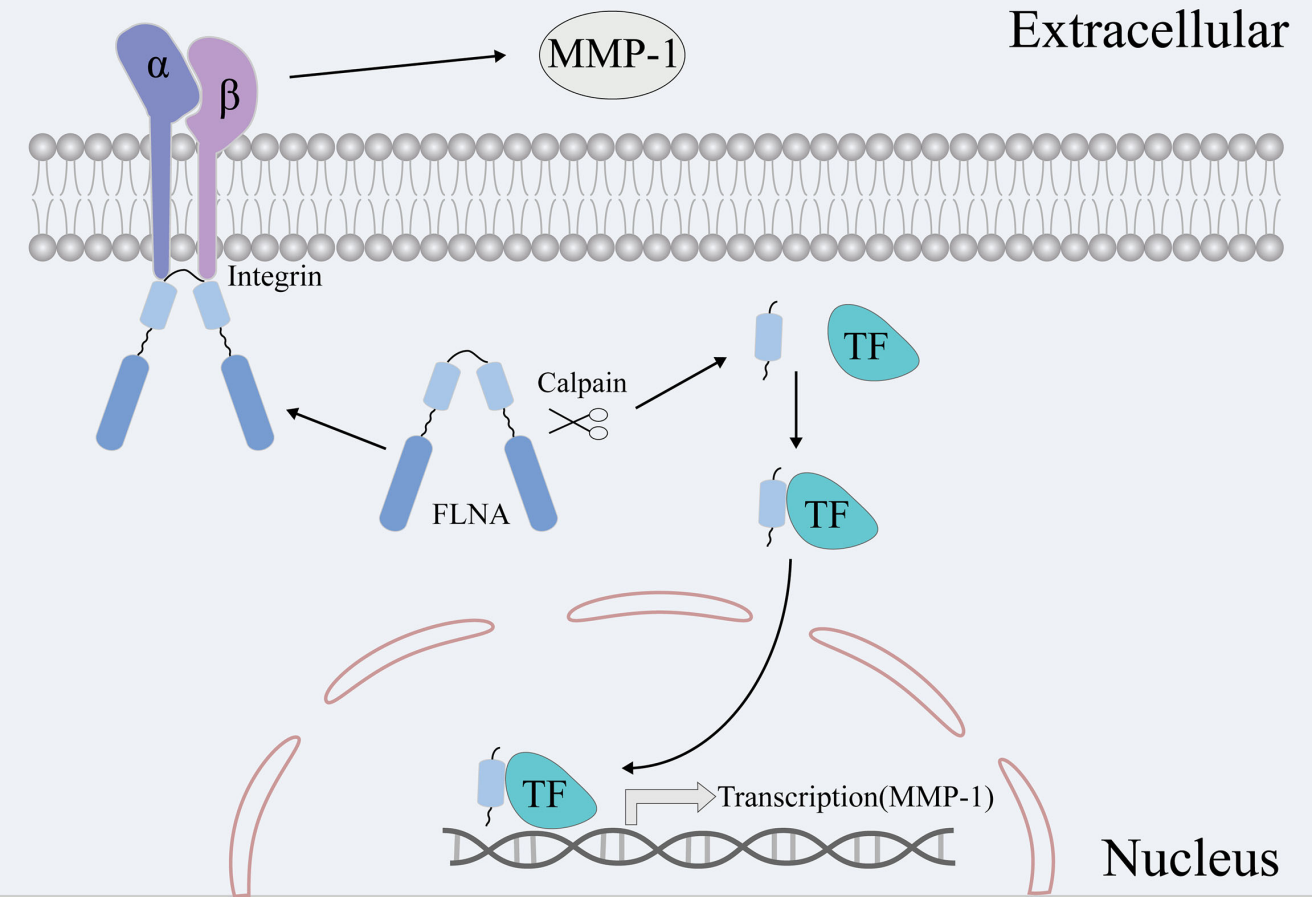
Two hypotheses are that FLNA promotes breast cancer metastasis *via* MMP-1. On the one hand, FLNA can affect the expression of MMP-1 by affecting the transport of transcription factors. On the other hand, FLNA can affect the activity and content of MMPs *via* the signaling route of ITGB1-MMPs.

In addition, we conducted preliminary research to investigate the possible mechanism underlying FLNA-regulated metastasis. We monitored the expression changes of four common EMT markers vimentin, β-catenin, Slug, and ZO-1 proteins after FLNA knockout. Previously, vimentin was reported to promote tumor metastasis through positive regulation of Axl (AXL Receptor Tyrosine Kinase) in breast cancer (42). However, we did not find any significant changes in these EMT phenotypic proteins after FLNA knockout. We considered that there might exist an alternative route like ECM, to promote tumor metastasis other than the EMT. Matrix metalloproteinases (MMPs) are a group of calcium-dependent zinc-containing endopeptidases, which mainly function in degrading ECM. MMP-1 is a ubiquitously expressed collagenase in ECM that can degrade type I, II, and III collagen (43). In this study, we found that knockout of FLNA significantly reduced the expression of MMP-1 but did not affect the other two ECM members MMP-2 and MMP-9. However, how FLNA regulates MMP-1 has not been fully elucidated. Bandaru et al. found that FLNA-C can be cleaved off by calpain to stimulate adaptive angiogenesis by transporting multiple transcription factors into the nucleus (44). Here, we found FLNA expressed in both the nucleus and cytoplasm of TNBC cell MDA-MB-231. Therefore, we speculated that FLNA-C might act as a transcription factor and directly or indirectly promote the expression of MMP-1 mRNA. Alternatively, prior works also found that FLNA could physically interact with integrin beta-1 (ITGB1) (45). ITGB1 can bind to various ECM components, which participate in multiple extracellular effects such as adhesion, ECM degradation, and cell invasion (46). Rizwan et al. found that stimulation of ITGB1 resulted in higher MMP activities in metastatic cancer cells (47). Accordingly, we monitored the expression of MMP-1, MMP-2, and MMP-9 in response to FLNA knockouts. The results manifested that only MMP-1 was significantly repressed in FLNA knockout cells. Previously, several works have suggested MMP-1 as a promoter of metastasis. For instance, overexpression of MMP-1 could promote the growth of xenograft tumors and the formation of brain metastasis (48). MMP-1 combined with ADAMTS1 can activate osteoclast differentiation by modulating the bone microenvironment in favor of osteoclastogenesis, to promote breast cancer bone metastasis (49). In summary, we speculate that FLNA likely promotes breast cancer metastasis in two different ways (**Figure 7**). FLNA-C interferes with the nucleo-cytoplasmic transportation of transcription factors to regulate MMP-1 expression, or FLNA regulates MMP-1 activities *via* interacting with the ITGB1-mediated signaling. To validate the mechanisms, extensive studies are desired in the future.

## Material and Methods

### Patients and Specimens

In this study, a cohort of nine breast cancer patients was recruited from the Cancer Hospital of Harbin Medical University. The study was approved by the Ethics Committee of Cancer Hospital of Harbin Medical University and Xiang’an Hospital of Xiamen University (XAHLL2020013) and abided by the Declaration of Helsinki principles. All patients were confirmed with recurrence of breast cancer, and the recurrent tumors were locoregional metastases (chest wall). The medical information of patients was briefly summarized in **Table 1**, and the individual information was replaced by anonymous digital codes. For every member in the cohort, paired tissue samples of the primary tumor and recurrent tumor were collected by surgery operation. The tumor tissues were then routinely formalin-fixed and paraffin-embedded (FFPE).

### DNA Extraction and Whole-Exome Sequencing

For each tissue sample, 3–5 µg of genomic DNA was applied for quality control, and its integrity was checked by the agarose electrophoresis. The whole exome was captured using the MGIEasy Exome Library Prep Kit (BGI, Shenzhen, China), and the library for sequencing was prepared according to the manufacturer’s instructions. The WES was performed by the Beijing Genome Institute (BGI, Shenzhen, China) using the BGIseq-500 platform in a 100-base pair (bp) paired-end mode.

### Exome Data Preprocessing, Variant Calling, and Variant Annotation

Before variant calling, the quality control of the exome data was conducted by FastQC (v.0.11.9, https://www.bioinformatics.babraham.ac.uk/projects/fastqc/) and Trimmomatic (v.0.39; parameters: LEADING = 5, TRAILING = 5, SLIDINGWINDOW:5:20, MINLEN = 50) (50) to remove adapter sequences and discard low-quality reads. The clean reads were mapped to the human reference genome (GRCh38.p13) using the Burrows–Wheeler Aligner (BWA, v.0.7.17; parameters: mem -t 4 -M -R) (51). The Genome Analysis Toolkit (GATK, v.4.1.2.0) (52) and Samtools (v.1.9) (53) were used for basic processing, duplicate marking, and base quality score recalibration (BQSR). Calling of somatic mutations was conducted with GATK Mutect2 (default parameters). The variants were further annotated with ANNOVAR (v2019sep29) (54). The datasets produced by this study were available in the Genome Variation Map portal repository at the following URL: https://ngdc.cncb.ac.cn/gvm/(accession number: GVM000287).

### Determination of Deleterious Variants

The deleterious variants for recurrent tumors were determined by satisfying several criteria: 1) the variant genotype was supported by a sequencing depth of >10. 2) Only four types of non-synonymous mutations at the exon region were involved in this study, including SNV, frameshift indel, non-frameshift indel, and stopgain and stoploss. 3) The occurrence of mutation in the Eastern Asian population was ≤1% as recorded in the ExAC_EAS database (55). 4) The variant was deleterious to protein. The deleteriousness of these non-synonymous variants was evaluated with multiple tools by different variant types. For SNVs, 15 tools were used to quantify the deleteriousness, including SIFT (56), Polyphen-2 HDIV (57), Polyphen-2 HVAR (57), LRT (58), MutationTaster (59), MutationAssessor (60), FATHMM (61), PROVEAN (62), VEST3 (63), MetaSVM (64), MetaLR (64), M_CAP (65), CADD (66), FATHMM-MKL (67), and fitCons (68). The variants were taken as deleterious variants if they were predicted pathogenic by more than twelve tools. For variants of frameshift Indel and stopgain, the deleteriousness was mainly assessed by checking the haploinsufficiency in the clinGen database (69). In addition, VEST-Indel (70) was also adopted to evaluate the deleteriousness of frameshift Indel and non-frameshift Indel mutations. The mutations with VEST Score ≥0.85 and VEST p-value ≤0.01 were considered as deleterious mutations. All stoploss variants were retained, as they were obviously harmful by adding part of a protein sequence.

### Survival Analysis

The survival analysis was conducted based on the database (71), which included 7,830 unique samples from 55 Gene Expression Omnibus (GEO) independent datasets to assess the impact of gene expression on breast cancer metastasis. Accordingly, overall 392 TNBC samples were involved in this analysis. The survival analysis was performed with the Kaplan–Meier Plotter web server (71).

### Cell Culture

All cell lines (including the normal breast mammary epithelial cell line MCF-10A, luminal A breast cancer cell lines MCF-7 and T-47D, luminal B breast cancer cell line BT-474, TNBC cell lines MDA-MB-231 and BT-549, and HER2+ cell line SK-BR-3) were purchased from the Type Culture Collection of the Chinese Academy of Sciences (Shanghai, China). MCF-10A were grown in MEGM kit (Lonza/Clonetics, CC-3150) with cholera toxin (Sigma, St. Louis, MO, USA; C8052) of 100 ng/ml. MCF-7 were grown in MEM (GIBCO, Grand Island, NY, USA; 41500034) with NaHCO_3_ 1.5 g/L, sodium pyruvate 0.11 g/L, and 0.01 mg/ml of bovine insulin. T47D and SK-BR-3 were grown in DMEM (GIBCO by Life Technologies, C11995500BT). BT474 was grown in Roswell Park Memorial Institute (RPMI) 1640 (GIBCO by Life Technologies, C11875500BT). MDA-MB-231 and MDA-MB-549 were grown in DMEM. All cell culture media were supplemented with 10% fetal bovine serum (FBS; GIBCO, 42A0378K) and 1% penicillin/streptomycin (GIBCO, 15140122). All cells were grown at 37°C and 5% CO_2_.

### Gene Knockout With CRISPR/Cas9 Technology

CRISPR/cas9 plasmid was synthesized by the Jikai Gene Company (Shanghai, China). The GV392 CRISPR-Cas9 vector had three gene-specific regions of the guide RNA (gRNA) sequences. The three gRNA sequences for FLNA were as follows: target 1: 5′-CACCGGCCCGTTACCAATGCGCGAG-3′, target 2: 5′-CACCGCGAGGTGACGGGGACTCATA-3′, and target 3: 5′-CACCGGAAGCGGGCAGAGTTCACTG-3′. The sequence 5′-CGCTTCCGCGGCCCGTTCAA-3′ of empty plasmid was used for NC (FLNA/NC). Transfection experiments were carried out in six-well plates. When the cell confluence reached 30%~40%, the transfection solution was added (*V* = *MOI* × *Cell number*/*Virus concentration*). After 24 h, stable FLNA knockout of MDA-MB-231 cells was obtained with 1 μg/ml of puromycin selection. FLNA knockout efficiency was evaluated by Western blot.

The overexpression MMP-1 plasmid was synthesized by the Jikai Gene Company (Shanghai, China), and it was anti-Blasticidin S. The sequencing results after successful plasmid construction are been shown in **Supplementary Materials 3**. After 24 h of infection, 5 ng/ml of Blasticidin S (Solarbio, Beijing, China; B9300) was added to select the overexpressing MMP-1 cells. The overexpression efficiency was verified by RT-qPCR and Western blot.

### Western Blot Analysis and Antibodies

The cells were fully lysed with RIPA (Lablead, Beijing, China; R1090), and the protein concentration was detected by bicinchoninic acid (BCA) kit (YEASEN, Shanghai, China; 20201ES76). The supernatant was then treated with 1/4 volume of 5× SDS-PAGE (YEASEN, 20315ES05), and cooked at 100°C for 10 min. Because the FLNA protein was large in molecular weight (280 kDa), gels were transferred onto polyvinylidene difluoride (PVDF) membranes (Millipore, Billerica, MA, USA; R1DB96261) at 250 mA for 3 h. The other protein transfer conditions were 80 V, 1.5 h. The primary antibody was incubated at 4°C overnight, and the secondary antibody was incubated at room temperature for 1 h. The details of antibodies are presented in **Supplementary Table 1**. To analyze the pictures, ImageJ was chosen.

### Quantitative Real-Time PCR (RT-qPCR)

The cells were fully lysed with TRIzol (ambion, Austin, TX, USA; 210805) to extract total RNA. Genomic DNA was removed, and the mRNAs were reverse transcribed into cDNA using Takara reverse transcription kit (Takara, Mountain View, CA, USA; RR047A). The PCR was conducted in a 20-μl system, including 2 μl of cDNAs, along with 0.4 μl of forward and reverse primers, 10 μl of SYBR (YEASEN, 11201ES03), and 7.2 μl of water. The specific primers for target RNA detection are given in **Supplementary Table 2**. Relative expression of each target gene was normalized to GAPDH mRNA level and calculated with the 2^−ΔΔCt^ method (72).

### Cell Proliferation

Cells were seeded onto 96-well (3 × 10^3^ cells/well) plates. Before measuring the optical density (OD), the cells were incubated with 10 μl/well of Cell Counting Kit-8 (CCK-8) (APEXBIO, Houston, TX, USA; K1018320180830) for 2 h. The OD value was measured at 450-nm spectrum by intervals of 0, 24, 48, and 72 h. The cell growth curve was drawn according to the OD value. *Cell growth rate* = (*control group OD* − *experimental group OD*)/*control group OD* × 100%.

### Cell Movement, Migration, and Invasion

#### Wound-Healing Assay

The wound-healing assay was initiated with 1 × 10^6^ cells/well in the six-well plate. When the cell conference was greater than 95% or just full, a straight line was drawn in the hole. Then the cells were continuously cultured in the serum-free medium to reduce the effect of cell proliferation on wound healing. The scratch changes were recorded by taking photos at 0, 12, 24, 36, and 48 h. The scratch area at each time point is defined with ImageJ by setting the parameter of *Wound-healing percentage* = (*Initial area* − *each time point area*)/*Initial area* × 100%.

#### Migration Assay

The cells were starved with the serum-free medium for 8 h and inoculated into transwell chambers. Each upper chamber was seeded with 2 × 10^4^ cells in 100 μl of serum-free medium (3.5 × 10^4^ cells of overexpressing MMP-1 were seeded into the upper chamber). A total of 800 μl of complete medium containing 10% FBS was added to the lower chamber. After 24 h, the cells were fixed with 4% paraformaldehyde (PFA; Biosharp, anhui, China, 71041800) and stained with crystal violet (Solarbio, G1063), and the upper cells were carefully wiped off with a cotton swab. Three visual fields were randomly selected to take photos and count under the microscope.

#### Invasion Assay

Cells were starved for 8 h before planking. Matrix glue measuring 90 μl (300 ng/ml) was to the upper chamber before plating 3 × 10^4^ cells in each upper chamber (4.5 × 10^4^ cells of overexpressing MMP-1 were seeded into the upper chamber). All the upper chambers were added with 100 μl of serum-free medium, whereas the lower chamber was added with a medium containing 10% FBS. After 24 h, the cells were fixed and stained, and three visual fields were randomly selected under the microscope for photographing and counting.

### Immunofluorescence

The cells were fixed with 4% PFA for 30 min, permeabilized with 0.5% Triton (Beyotime, Shanghai, China; ST795) for 10 min, blocked with 5% bovine serum albumin (BSA; YEASEN, 36101ES25) for 30 min (slow shaking), and incubated with primary FLNA antibody at 4°C overnight and secondary antibody (FITC-AffiniPure Goat Anti-Rabbit IgG) at room temperature for 1 h. The antibodies and their corresponding dilution are given in **Supplementary Table 2**. One milliliter of 1× phalloidin (YEASEN, 40734ES75) into each culture dish and dyed at room temperature for 60 min, especially avoiding light. Subsequently, 3–4 drops of DAPI (YEASEN, 40728ES10) were added to each dish and incubated at room temperature for 5 min. The localization of FLNA and cell morphology were observed under the microscope and photographed. Phalloidin was used for F-actin staining as pink. FLNA was stained green with fluorescently conjugated secondary antibody. DAPI stained the nucleus blue.

### Xenograft Model

All procedures of the mouse model were approved by the Xiamen University (AP: XMULAC20200119) and conformed to the guidelines for the care and maintenance of laboratory animals. Breast cancer cells (5 × 10^6^ cells/mouse) were injected into the fourth pair of mammary glands on the right side of 6-week-old female Balb/c nude mice according to the above groups (73, 74). There were 5 mice in each group. The length and width of the tumor *in situ* were monitored with a vernier caliper. The calculation formula of tumor volume in athymic nude mice is V = 0.5 * Length * Width^2^ (mm^3^) (W, smaller diameter; L, larger diameter) as described previously (75). After 4 weeks, the mice were sacrificed, and the liver, kidney, lung, and brain of mice were collected to evaluate the metastatic state.

### H&E Stain and Immunohistochemistry

The tissue sections were dewaxed in xylene and hydrated in alcohol. The nucleus and cytoplasm were stained by hematoxylin (Beyotime, C0105S) and eosin, respectively. The stained tissues were dehydrated and sealed, and they were observed and image-captured under a microscope.

The immunohistochemical assay was performed on FFPE sections of xenograft mouse tissues. Tumor sections measuring 5 μm were incubated with primary antibody at 4°C overnight and secondary antibody at room temperature for 2 h. Subsequently, all fields were observed under light microscopy. ImageJ was used to calculate the integrated OD (IOD), the distribution area of IHC staining images, and the average OD (AOD). *AOD* = *IOD*/*Area*.

### Statistical Analysis

GraphPad Prism 8.0.1 software was used for statistical analyses. All data were presented as mean ± SD of at least three independent experiments. One-way ANOVA was selected for more than two groups. *p < 0.05, **p < 0.01, or ****p < 0.0001 was labeled for statistical significance.

## Data Availability Statement

The datasets presented in this study can be found in online repositories. The names of the repository/repositories and accession number(s) can be found in the article/**Supplementary Material**.

## Ethics Statement

The animal study was reviewed and approved by Xiamen University (AP: XMULAC20200119). Written informed consent was obtained from the individual(s) for the publication of any potentially identifiable images or data included in this article.

## Author Contributions

JZ designed and performed the experiments and analyzed the data. LW analyzed the sequencing data of clinical samples. JZ and LW drafted the manuscript and made the tables. XK designed and supervised all the experiments, participated in the revision of manuscript. ZJ provided guidance for sequencing data analysis and revised the manuscript. PX collected clinical samples. LY participated in animal experiments and revised the manuscript. All authors listed have made a substantial, direct, and intellectual contribution to the work and approved it for publication.

## Funding

This work was supported by the Young and Middle-aged Talents Training Program of Fujian Provincial Health Commission (2020GGB062), Natural Science Foundation of Fujian Province (2019J01012), and Scientific Research Foundation for Advanced Talents, Xiang’an Hospital of Xiamen University (PM201809170014).

## Conflict of Interest

The authors declare that the research was conducted in the absence of any commercial or financial relationships that could be construed as a potential conflict of interest.

## Publisher’s Note

All claims expressed in this article are solely those of the authors and do not necessarily represent those of their affiliated organizations, or those of the publisher, the editors and the reviewers. Any product that may be evaluated in this article, or claim that may be made by its manufacturer, is not guaranteed or endorsed by the publisher.
