# Supplementary material for: Filamin A Is a Potential Driver of Breast Cancer Metastasis via Regulation of MMP-1
Source: Front Oncol. 2022 Mar 11;12:836126. doi: 10.3389/fonc.2022.836126 (PMC8962737; doi:10.3389/fonc.2022.836126)
Supplement: Supplementary file 1 [file DataSheet_1.docx]

**Full Name and Main Cellular Function**

**1. Filamin A (FLNA):** The protein encoded by this gene is an actin-binding protein that crosslinks actin filaments and links actin filaments to membrane glycoproteins.

**2. Matrix Metallopeptidase 1 (MMP-1):** This gene encodes a member of the peptidase M10 family of matrix metalloproteinases (MMPs). Proteins in this family are involved in the breakdown of extracellular matrix in normal physiological processes, such as embryonic development, reproduction, and tissue remodeling, as well as in disease processes, such as arthritis and metastasis.

**3. BRCA1 DNA Repair Associated (BRCA1):** This gene encodes a 190 kD nuclear phosphoprotein that plays a role in maintaining genomic stability, and it also acts as a tumor suppressor.

**4. BRCA2 DNA Repair Associated (BRCA2):** Inherited mutations in BRCA1 and this gene, BRCA2, confer increased lifetime risk of developing breast or ovarian cancer. Both BRCA1 and BRCA2 are involved in maintenance of genome stability, specifically the homologous recombination pathway for double-strand DNA repair.

**5. Partner And Localizer Of BRCA2 (PALB2):** This gene encodes a protein that may function in tumor suppression. This protein binds to and colocalizes with the breast cancer 2 early onset protein (BRCA2) in nuclear foci and likely permits the stable intranuclear localization and accumulation of BRCA2.

**6. Tumor Protein P53 (TP53):** This gene encodes a tumor suppressor protein containing transcriptional activation, DNA binding, and oligomerization domains.

**7. Interleukin 1 Beta (IL-1β):** This cytokine is an important mediator of the inflammatory response, and is involved in a variety of cellular activities, including cell proliferation, differentiation, and apoptosis.

**8. Phosphatase And Tensin Homolog (PTEN):** The protein encoded by this gene is a phosphatidylinositol-3,4,5-trisphosphate 3-phosphatase. It negatively regulates intracellular levels of phosphatidylinositol-3,4,5-trisphosphate in cells and functions as a tumor suppressor by negatively regulating AKT/PKB signaling pathway.

**9. Cartilage Oligomeric Matrix Protein (COMP):** The protein encoded by this gene is a noncollagenous extracellular matrix (ECM) protein.

**10. Forkhead Box O3 (FOXO3):** This gene likely functions as a trigger for apoptosis through expression of genes necessary for cell death.

**11. Heat Shock Protein Family A (Hsp70) Member 2 (HSPA2):** Diseases associated with HSPA2 include Crohn's Disease and Varicocele. Among its related pathways are Meiosis and Proteolysis Role of Parkin in the Ubiquitin-Proteasomal Pathway.

**12. Inositol 1,4,5-Trisphosphate Receptor Type 3 (ITPR3):** This gene encodes a receptor for inositol 1,4,5-trisphosphate, a second messenger that mediates the release of intracellular calcium. The receptor contains a calcium channel at the C-terminus and the ligand-binding site at the N-terminus.

**13. Phosphoinositide-3-Kinase Regulatory Subunit 2 (PIK3R2):** The protein encoded by this gene is a regulatory component of PI3K.

**14. Neurofibromin 1 (NF1):** This gene product appears to function as a negative regulator of the ras signal transduction pathway.

**15. GATA Binding Protein 3 (GATA3):** This gene encodes a protein which belongs to the GATA family of transcription factors. The protein contains two GATA-type zinc fingers and is an important regulator of T-cell development and plays an important role in endothelial cell biology.

**16. Tight junction protein ZO-1 (ZO-1):** This gene encodes a member of the membrane-associated guanylate kinase (MAGUK) family of proteins, and acts as a tight junction adaptor protein that also regulates adherens junctions.

**17. Zinc finger protein SNAI2 (Slug):** Transcriptional repressor that modulates both activator-dependent and basal transcription.

**18. β- Catenin:** The protein encoded by this gene is part of a complex of proteins that constitute adherens junctions (AJs).

**19. Vimentin:** The encoded protein is responsible for maintaining cell shape and integrity of the cytoplasm, and stabilizing cytoskeletal interactions.

**20. Matrix Metallopeptidase 2 (MMP-2):** This gene is a member of the matrix metalloproteinase (MMP) gene family, that are zinc-dependent enzymes capable of cleaving components of the extracellular matrix and molecules involved in signal transduction.

**21. Matrix Metallopeptidase 9 (MMP-9):** The enzyme encoded by this gene degrades type IV and V collagens.

**22. Epidermal Growth Factor Receptor (EGFR):** The protein encoded by this gene is a transmembrane glycoprotein that is a member of the protein kinase superfamily. This protein is a receptor for members of the epidermal growth factor family.

**23. Integrin Subunit Beta 1 (ITGB1):** This gene encodes a beta subunit of integrins.Integrin family members are membrane receptors involved in cell adhesion and recognition in a variety of processes including embryogenesis, hemostasis, tissue repair, immune response and metastatic diffusion of tumor cells.
