# Supplementary material for: Filamin A Is a Potential Driver of Breast Cancer Metastasis via Regulation of MMP-1
Source: Front Oncol. 2022 Mar 11;12:836126. doi: 10.3389/fonc.2022.836126 (PMC8962737; doi:10.3389/fonc.2022.836126)
Supplement: Supplementary file 2 [file DataSheet_2.docx]

**Supplementary 3：**

**Sequencing results of MMP-1 overexpression plasmid**

The green marker GCA synonymous mutation is GCG; TAC synonymous mutation is TAT.

GTTTTTGGCTTTTTTGTTAGACGAAGCTTGGGCTGCAGGTCGACTCTAGAGGATCCCGCCACCATGCACAGCTTTCCTCCACTGCTGCTGCTGCTGTTCTGGGGTGTGGTGTCTCACAGCTTCCCAGCGACTCTAGAAACACAAGAGCAAGATGTGGACTTAGTCCAGAAATACCTGGAAAAATACTACAACCTGAAGAATGATGGGAGGCAAGTTGAAAAGCGGAGAAATAGTGGCCCAGTGGTTGAAAAATTGAAGCAAATGCAGGAATTCTTTGGGCTGAAAGTGACTGGGAAACCAGATGCTGAAACCCTGAAGGTGATGAAGCAGCCCAGATGTGGAGTGCCTGATGTGGCTCAGTTTGTCCTCACTGAGGGGAACCCTCGCTGGGAGCAAACACATCTGACCTACAGGATTGAAAATTACACGCCAGATTTGCCAAGAGCAGATGTGGACCATGCCATTGAGAAAGCCTTCCAACTCTGGAGTAATGTCACACCTCTGACATTCACCAAGGTCTCTGAGGGTCAAGCAGACATCATGATATCTTTTGTCAGGGGAGATCATCGGGACAACTCTCCTTTTGATGGACCTGGAGGAAATCTTGCTCATGCTTTTCAACCAGGCCCAGGTATTGGAGGGGATGCTCATTTTGATGAAGATGAAAGGTGGACCAACAATTTCAGAGAGTACAACTTACATCGTGTTGCGGCTCATGAACTCGGCCATTCTCTTGGACTCTCCCATTCTACTGATATCGGGGCTTTGATGTACCCTAGCTACACCTTCAGTGGTGATGTTCAGCTAGCTCAGGATGACATTGATGGCATCCAAGCCATATATGGACGTTCCCAAAATCCTGTCCAGCCCATCGGCCCACAAACCCCAAAAGCGTGTGACAGTAAGCTAACCTTTGATGCTATAACTACGATTCGGGGAGAAGTGATGTTCTTTAAAGACAGATTCTACATGCGCACAAATCCCTTCTACCCGGAAGTTGAGCTCAATTTCATTTCTGTTTTCTGGCCACAACTGCCAAATGGGCTTGAAGCTGCTTACGAATTTGCCGACAGAGATGAAGTCCGGTTTTTCAAAGGGAATAAGTACTGGGCTGTTCAGGGACAGAATGTGCTACACGGATACCCCAAGGACATCTACAGCTCCTTTGGCTTCCCTAGAACTGTGAAGCATATCGATGCTGCTCTTTCTGAGGAAAACACTGGAAAAACCTACTTCTTTGTTGCTAACAAATACTGGAGGTATGATGAATATAAACGATCTATGGATCCAGGTTATCCCAAAATGATAGCACATGACTTTCCTGGAATTGGCCACAAAGTTGATGCAGTTTTCATGAAAGATGGATTTTTCTATTTCTTTCATGGAACAAGACAATATAAATTTGATCCTAAAACGAAGAGAATTTTGACTCTCCAGAAAGCTAATAGCTGGTTCAACTGCAGGAAAAATACCGGTATGGACTACAAGGATGACGATGACAAGGATTACAAAGACGACGATGATAAGGACTATAAGGATGATGACGACAAATGAGCTAGCCTGTGGAATGTGTGTCAGTTAGGGTGTGGAAAGTCCCCAGGCTCCCCAGCAGGCAGAAGTATGCAAAGCATGCATCTCAATTAGTCAGCAACCAGGTGTGGAAAGTCCCCAGGCTCCCCAGCAGGCAGAAGTATGCAAAGCATGCATCTCAATTAGTCAGCAACCATAGTCCCGCCC
