# Supplementary material for: Filamin A Is a Potential Driver of Breast Cancer Metastasis via Regulation of MMP-1
Source: Front Oncol. 2022 Mar 11;12:836126. doi: 10.3389/fonc.2022.836126 (PMC8962737; doi:10.3389/fonc.2022.836126)
Supplement: Supplementary file 3 [file Table_1.docx]

**Supplementary Table 1. Antibodies used in the study**

| Antibody | Vendors | Catalogue No. | Applications | Dilution |
| --- | --- | --- | --- | --- |
| FLNA | abcam | EP2405Y | WB | 1:1000 |
|  |  |  | IF | 1:200 |
|  |  |  | IHC | 1:100 |
| MMP-1 | abcam | EP1249Y | WB | 1:1000 |
| MMP-1 | abcam | EP1247Y | IHC | 1:100 |
| MMP-2 | CST | 40994S | WB | 1:1000 |
| MMP-9 | CST | 13667S | WB | 1:1000 |
| GATA3 | CST | 5852S | IHC | 1:1600 |
| GAPDH | CST | 5174S | WB | 1:1000 |
| Anti-rabbit IgG | CST | 7074S | WB | 1:1000 |
| Anti-mouse IgG | CST | 7076S | WB | 1:1000 |
| FITC-AffiniPure Goat Anti-Rabbit IgG | YEASEN | 33107ES60 | IF | 1:25 |
| Vimentin | CST | 5741S | WB | 1:1000 |
| β-catenin | CST | 8480S | WB | 1:1000 |
| Slug | CST | 9585S | WB | 1:1000 |
| ZO-1 | CST | 13663S | WB | 1:1000 |
| Ki-67 | CST | 9449S | IHC | 1:100 |
