# Supplementary material for: Filamin A Is a Potential Driver of Breast Cancer Metastasis via Regulation of MMP-1
Source: Front Oncol. 2022 Mar 11;12:836126. doi: 10.3389/fonc.2022.836126 (PMC8962737; doi:10.3389/fonc.2022.836126)
Supplement: Supplementary file 4 [file Table_2.docx]

**Supplementary Table 2. List of primers used in the study.**

| Gene name | Sequence |
| --- | --- |
| MMP-1 | Forward: GGGGCTTTGATGTACCCTAGC |
|  | Reverse: TGTCACACGCTTTTGGGGTTT |
| MMP-2 | Forward: GATACCCCTTTGACGGTAAGGA |
|  | Reverse: CCTTCTCCCAAGGTCCATAGC |
| MMP-9 | Forward: AGACCTGGGCAGATTCCAAAC |
|  | Reverse: CGGCAAGTCTTCCGAGTAGT |
| GAPDH | Forward: GGAGCGAGATCCCTCCAAAAT |
|  | Reverse: GGCTGTTGTCATACTTCTCATGG |
